# Assessing language background and experiences among heritage bilinguals

**DOI:** 10.3389/fpsyg.2022.993669

**Published:** 2022-10-06

**Authors:** Alessandra Macbeth, Natsuki Atagi, Jessica L. Montag, Michelle R. Bruni, Christine Chiarello

**Affiliations:** ^1^Department of Psychology, Azusa Pacific University, Azusa, CA, United States; ^2^Department of Child and Adolescent Studies, California State University, Fullerton, Fullerton, CA, United States; ^3^Department of Psychology, University of Illinois, Urbana-Champaign, Champaign, IL, United States; ^4^Department of Psychology, University of California, Riverside, Riverside, CA, United States

**Keywords:** heritage bilinguals, electronically activated recorder, self-report questionnaires, language assessments, multilingual naming test, verbal fluency

## Abstract

The language backgrounds and experiences of bilinguals have been primarily characterized using self-report questionnaires and laboratory tasks, although each of these assessments have their strengths and weaknesses. The Electronically Activated Recorder (EAR), an audio recording device, has recently become more prominent as a method of assessing real-world language use. We investigated the relationships among these three assessment tools, to understand the shared variance in how these measures evaluated various aspects of the bilingual experience. Participants were 60 Southern California heritage bilingual college students who spoke a variety of heritage languages and began to learn English between the ages of 0-to 12-years. Participants completed both self-report and laboratory-based measures of language proficiency and use, and they wore the EAR for 4  days to capture representative samples of their day-to-day heritage language (HL) use. The results indicated that self-reported HL use and English age of acquisition were significant predictors of real-world language use as measured by the EAR. In addition, self-reported HL proficiency and laboratory-based HL proficiency, as measured by verbal fluency, were mutually predictive. While some variability was shared across different assessments, ultimately, none of the measures correlated strongly and each measure captured unique information about the heritage bilingual language experience, highlighting the dissociation between language experience measured at a single point in time and an accumulated life history with a heritage language. These findings may provide guidance for bilingualism researchers about which assessment tool, or combination of tools, may be best for their specific research questions.

## Introduction

Bilinguals regularly encounter diverse linguistic experiences in their day-to-day lives, as a function of the ability to speak their two languages in different contexts and with different interlocutors. Although much of the psycholinguistic research over the past two decades has treated bilingualism categorically (but see also Gollan et al., 2011), there has been a recent push to consider bilingualism as a continuous spectrum of dynamic experiences that uniquely affect cognition and the neural indices of brain structure and function over time (Kaushanskaya and Prior, 2015; Luk and Pliatsikas, 2016; Takahesu Tabori et al., 2018; de Bruin, 2019; DeLuca et al., 2019). This updated perspective acknowledges that a given sample of bilinguals could vary widely with respect to their levels of proficiency, frequency of use, and experience with their given languages. As such, it has become increasingly important that current studies appropriately characterize the bilingual experiences of their participants.

The two most common methods employed in psycholinguistic research on bilingualism today are self-report questionnaires of language background and laboratory tests of language proficiency. However, neither measure provides objective insight into day-to-day bilingual language use. The inclusion of such real-world data in this field is important, because it may provide unique information about a bilingual’s language experience and proficiency beyond what self-report or laboratory tasks can tell us. To address this issue and capture variability in day-to-day bilingual language use, the present study employed the Electronically Activated Recorder (EAR, Mehl et al., 2001; Mehl, 2017) to record real-world language use among a linguistically diverse sample of heritage bilingual undergraduates from Southern California. Studying heritage speakers represents a unique opportunity to examine diversity among adult bilinguals.

Heritage bilinguals are individuals who typically learned a home language from birth (i.e., their heritage language) and learned the community language through immersion during childhood (Rothman, 2009; Montrul, 2016), oftentimes when they started school. Although heritage bilinguals constitute over 75% of the bilingual population in the United States (American Academy of Arts and Sciences Commission on Language Learning, 2017), psycholinguistic research on bilingualism—even in the United States—has largely overlooked this population. Heritage bilinguals are a diverse group, who may or may not have had any formal education in their heritage language (Carreira and Kagan, 2011), tend to be more dominant in the community language (e.g., Rothman and Treffers-Daller, 2014; Sanz and Torres, 2018), and may vary widely in the skill with which they use their heritage language (e.g., Kupisch and Rothman, 2018; Polinsky, 2018). For instance, although heritage bilinguals often *understand* their heritage language “very well” (American Academy of Arts and Sciences Commission on Language Learning, 2017), their skill in *speaking* the heritage language may range from minimal to being highly proficient in the language (e.g., Valdés, 2001; Polinsky and Kagan, 2007). Thus, heritage bilinguals are characterized by a wide range of language histories, language experiences, and language skills.

To better describe and understand the range of lived language experiences of heritage bilinguals, or other bilingual speakers, we must describe and document these ranges of experiences. Existing methods largely focus on early experience with language, such as the age at which an individual first began learning a language, or a description of the languages spoken in the home and at school during childhood (e.g., Surrain and Luk, 2019). These experiences, which aim to describe early and habitual experiences with a language, may be quite different from current patterns of language experience. For example, individuals with greater amounts of early childhood experience with a language may or may not maintain that experience into adulthood, and individuals who were less deeply immersed in a language through childhood may or may not use that language frequently in adulthood (e.g., Valdés, 2001; De Houwer, 2021). Current patterns of language use may be a characteristic of the bilingual experience that is both distinct from childhood language experience as well as various measures of language proficiency. Measuring current patterns of language use, and the frequencies and contexts in which a speaker uses their multiple languages, may be a way to further develop an understanding of the wide range of bilingual experiences.

Measuring current patterns of language use also allows us to document the prevalence of specific behaviors, such as code-switching. Code-switching, or the use of two or more languages within an utterance or conversation (Gumperz, 1977), is a linguistic phenomenon common in some bilingual speech that has been found to be strongly correlated with measures of language entropy or diversity in language use (Kałmała et al., 2022). Although once believed to indicate a bilingual speaker’s “confusion” between their two languages, code-switching occurs systematically and in fact demonstrates high proficiency in both languages (e.g., Poplack, 1980; Bentahila and Davies, 1983; Otheguy and Lapidus, 2003). Moreover, code-switching may reflect various cognitive strategies that bilinguals use to effectively produce speech (for review, see Beatty-Martínez et al., 2020). For example, code-switching may be a cognitive tool for bilinguals to produce hard-to-retrieve lexical items (e.g., Sarkis and Montag, 2021). Code-switching behavior therefore reflects an array of linguistic, pragmatic, and cognitive skill in bilinguals’ use of their two languages.

The present study had three goals. The first was to examine the relationships among common self-report language background items, laboratory tasks of language proficiency, and real-world language use (as measured by the EAR). Second, we studied how well self-report predicts day-to-day heritage language use, and vice versa, in a sample of 60 heritage bilinguals. Third, we examined all three measures (self-report, laboratory tasks, and actual, current language use) and the partial correlations that exist between them, to further investigate where variance is–or is not–shared among these three converging but distinct measures of the heritage bilingual language experience. We also report some exploratory analyses with self-reported and EAR-captured code-switching data, which might provide further insight into the ability of heritage bilinguals to accurately gauge their own language use. Ultimately, we hope that the findings reported herein can help future researchers decide which tool (or combination of tools) to use when assessing the language background and experiences of heritage bilinguals, depending on the types of questions they hope to answer.

## A brief overview of various language assessment tools

### Self-report measures

Surveys in which individuals report information about their language history, use, and proficiency are one of the most commonly used methods to assess language background. Current self-report measures that are commonly used to assess the language backgrounds of bilinguals, in particular their proficiency/use of each language, include the Language History Questionnaire (LHQ; Li et al., 2006, 2014, 2020), the Language Experience and Proficiency Questionnaire (LEAP-Q; Marian et al., 2007), and the Language and Social Background Questionnaire (LSBQ; Luk and Bialystok, 2013; Anderson et al., 2018)—all of which have been found to be valid and reliable measures of language backgrounds (Li et al., 2006; Marian et al., 2007; Luk and Bialystok, 2013). In particular, the LEAP-Q is thought to provide a fairly robust assessment of language proficiency, while the LSBQ is arguably the preferred self-report measure for those interested in language use (de Bruin, 2019). Such questionnaires are also generally quick and easy to administer.

Although these surveys often vary in their operationalizations of and the exact manner in which they ask about language history, use, and proficiency (see Kašćelan et al., 2021), there are commonalities in the types of questions asked. For example, to assess language history, surveys typically include questions about when (e.g., age of acquisition), how (e.g., immersion, formal education), and from whom (e.g., family, teachers) individuals learned each language in the past. To assess language use, surveys often include items about how frequently, in what contexts, and with whom individuals currently use each language and used each language in the past. And to assess language proficiency, surveys commonly ask individuals to rate their current level of fluency in speaking, understanding, reading, and/or writing in each of their languages. The combination of these questions is intended to provide insight into the ways in which a bilingual’s current language use and proficiency may differ from their past language use.

However, these assessment tools are not perfect; they often depend on ordinal measures (e.g., Likert scales), which can lead to seemingly arbitrary responses that are difficult to interpret. For example, two individuals with the same self-assigned Likert ratings could, in reality, have entirely different levels of proficiency/use, bringing into question the inherent utility of such measurements (Sechrest et al., 1996). Further, some commonly used questionnaires differ in the range of values on their scales. For example, the LEAP-Q and LSBQ have participants rate their proficiency on a scale of 0–10 (Marian et al., 2007; Anderson et al., 2018), whereas the LHQ utilizes a 1–7 scale (Li et al., 2020). In these cases, it is unclear whether participants would rate themselves equivalently on the two versions (e.g., Would one who rates themselves as 5/7 on English speaking proficiency when given the LHQ also assign themselves a 7/10 rating, a roughly equivalent score, on the LEAP-Q?). Although one proposed solution for creating consistency across such measures has been to develop a *Bilingualism Quotient* (Marian and Hayakawa, 2021), there is currently no single agreed upon way for language experience to be operationalized or measured (cf. Kałamała et al., 2022).

Additionally, self-report measures of second language (L2) proficiency/use may be swayed by participants’ own biases. Some young adults are prone to overestimation of their L2 proficiency/use (MacIntyre et al., 1997; Gollan et al., 2012), and individuals who are more anxious about their L2 abilities may be prone to underestimation of L2 proficiency/use (MacIntyre et al., 1997). Self-assessment of one’s language proficiency/use might also be skewed due to lack of interaction with an appropriate comparison group. For example, one might have an inflated sense of how proficient they are in a language if they have few native speakers in their local environments to compare themselves to (Blanche, 1988; Blanche and Merino, 1989). Thus, not only is the age of the participants important to consider when interpreting self-report measures of L2 proficiency/use, but the context in which participants use a language and the range of speakers with which they interact must also be considered.

Abundant evidence also suggests that individuals from different cultural backgrounds may interpret or respond to self-report questions in systematically different ways. Tomoschuk et al. (2019) found the way in which individuals from different cultural backgrounds respond to measures of proficiency can vary widely. Spanish-English bilinguals who rated themselves as highly proficient in Spanish (e.g., 7/7) scored lower on the Multilingual Naming Test (MINT; Gollan et al., 2012)–a laboratory task of language proficiency–than their Chinese-English bilingual counterparts who rated themselves equally highly on Chinese proficiency. Among participants who considered their proficiency in Spanish or Chinese low (e.g., 3/7), the opposite effect emerged: Chinese-English bilinguals’ MINT scores in Chinese were significantly lower than Spanish-English bilinguals’ MINT scores in Spanish. Such findings corroborate previous work by the same group showing that Chinese-English bilinguals are generally more accurate when self-reporting their non-English language proficiency than are Spanish-English bilinguals (Sheng et al., 2014). Additionally, Hoshino and Kroll (2008) found that despite similar lab-based English proficiency (measured by picture naming), Japanese-English bilinguals self-rated their English proficiency significantly lower than Spanish-English bilinguals. Taken together, these studies suggest that while self-report is a commonly used method in the psycholinguistics literature, culture and other participant characteristics contribute non-random error to the observed data.

### Laboratory tasks

One seemingly straightforward way to test the validity of participants’ self-report on language background measures would be to employ laboratory-based measures of language ability. Some common measures include picture naming tasks such as the Peabody Picture Vocabulary Test (PPVT; Dunn and Dunn, 1997), the Boston Naming Test (BNT; Kaplan et al., 1983), LexTALE (Lemhöfer and Broersma, 2012), and the MINT (Gollan et al., 2012), all of which assess vocabulary knowledge as a proxy for language proficiency and have been normed in multiple languages. Verbal fluency measures, which assess lexical knowledge, retrieval, and production as a proxy for language proficiency, are frequently employed as well (Gollan et al., 2002; Portocarrero et al., 2007; Lezak et al., 2012; Friesen et al., 2015). Past research has suggested that moderate correlations exist between self-reports of language background and these laboratory measures (Marian et al., 2007; de Bruin et al., 2017). However, less than half of the studies recently published in the psycholinguistic bilingualism field include a laboratory measure of language proficiency or fluency (Hulstijn, 2012; Surrain and Luk, 2019), and the majority of studies rely solely on self-report measures of language proficiency.

In addition, both the MINT and verbal fluency also have their own shortcomings. Measures of vocabulary such as the MINT overlook other critical components of language skill such as syntax or sentence production (Paap et al., 2017), and assume that the words used on the test are unbiased and indeed a good index of vocabulary size. Further, performance on verbal fluency tasks can be influenced by participant variables such as age or executive control abilities that are independent of linguistic knowledge (Friesen et al., 2015). Of course, neither of these tasks measure actual, real-world language use, which may differ from either laboratory tasks or the self-report measures.

### The EAR

The EAR–or Electronically Activated Recorder–is a free Android app that captures naturalistic data *via* audio snippets from a participant’s day-to-day life (Mehl et al., 2001; Mehl, 2017). Importantly, the language data captured by the EAR reflects spontaneous, current speech use (Mehl et al., 2012) - participants cannot track when the EAR is recording, so there are no expectancy effects regarding when or how to speak. By using this time sampling approach, the EAR provides insight into what language(s) a participant is using day-to-day, the environmental and linguistic contexts in which those languages are being used, and the frequency with which different languages are being spoken (Macbeth et al., 2022). The EAR can provide an objective measure of an individual’s frequency of language use, which is important for understanding an individual’s language habits and experiences.

Because the EAR measures language use, a characteristic commonly included in surveys of language background, we can use the EAR to determine how well self-report captures objective real-world behaviors and experiences, and to assess the validity of self-report data. One such study showed that participants are quite accurate at gauging how much they participate in behaviors such as listening to music, watching TV, or talking to others (Vazire and Mehl, 2008). In another study that compared the talkativeness of Mexican versus American individuals, cultural differences in the validity of self-report data emerged: Americans rated themselves as being more talkative and sociable, despite engaging in fewer conversations, spending less time with others, and talking less than the Mexican participants (Ramírez-Esparza et al., 2009). Similarly, a more recent study by Marchman et al. (2017) that used the LENA system^1^ found that Spanish-English bilingual parents systematically underestimated the use of their dominant language (Spanish) and overestimated the use of their less dominant language (English) in their self-report of child-directed speech in each language. Altogether, these findings suggest that collecting objective measures of language use can improve our interpretation of self-reported language use.

The field has developed many different means to capture aspects of an individual’s language history, current language use, and language proficiency. Each of these tools has advantages and disadvantages in their ease of implementation and interpretability of the data. In the present study, we propose that the EAR may be another tool to add to this list, and that observation of naturalistic language use outside the lab may be a useful means to better understand an individual’s current patterns of language use. We believe the recordings captured by the EAR may reflect a construct not well captured by existing tools—current patterns of language use need not align with past or childhood measures of language use nor with measures of language proficiency. Understanding current day-to-day patterns of language use as a qualitatively different construct may be helpful for understanding the diverse range of experiences that bilingual speakers encounter.

## The current study

In the current study, we examined how each of the three assessments used to measure heritage bilingual language abilities and use (self-report, laboratory tasks, and the EAR) are related and what shared variance is (or is not) captured among them. This will allow us to better understand the language backgrounds and day-to-day language experiences among our sample of heritage bilingual speakers.

Because we anticipated that our sample of heritage bilinguals would be highly proficient in English, we expected the most variability in heritage language (HL) proficiency and use. Further, English age of acquisition (AoA) is also important to consider: The age at which heritage bilinguals learned English might affect when and how often they use their HL (e.g., De Houwer, 2021). Moreover, AoA is commonly examined in psycholinguistic studies of bilingualism and is frequently related to language proficiency (e.g., Johnson and Newport, 1989; Birdsong, 1992; Flege et al., 1999; Hakuta et al., 2003). Therefore, the self-report variables that we focused on in the present study were HL proficiency, HL use, and English AoA. We chose laboratory tasks that are commonly used to assess language proficiency in the existing literature, so we administered both the verbal fluency and MINT to participants. Although both tasks measure productive language skill, the MINT is a standardized measure of English vocabulary that could be used with all participants, regardless of the HL spoken by participants. Finally, we measured actual day-to-day HL use *via* the EAR.

We expected all three assessment tools to be significantly correlated, but to differing extents depending on the primary construct being measured by each tool. These measures are not necessarily different assessments of a single underlying construct, but rather likely represent different constructs that may relate to each other in interesting ways. For example, the EAR is primarily a measure of language use, so we hypothesized that self-reported HL use would best predict EAR-based HL use, and vice versa. Likewise, laboratory-based measures such as verbal fluency and the MINT are generally used as assessments of language proficiency, so we expected that self-reported HL proficiency would be mutually predictive of performance on these tasks. Further, we were interested in how self-reported AoA would relate to these other measures. If a heritage bilingual acquired their second language (English) later in life, then we would expect them to be more proficient in their HL and use their HL more. We were interested in the shared (and unshared) variance captured by each of these different assessment tools and reported the partial correlations through a series of regression models to demonstrate where these measures overlapped, and where they did not. Finally, we explored self-report items related to code-switching in order to determine which one(s) best predicted actual code-switching as measured by the EAR and gauge how well heritage bilinguals can assess their own code-switching frequency. From these results, our hope is that we can begin to understand the relative contributions of each bilingualism measure and how they can collectively contribute to a comprehensive understanding of bilingual language experience.

## Materials and methods

### Participants

Our sample consisted of 60 heritage bilingual participants (38 women, 22 men, *M* = 19.25 years) from the University of California, Riverside, and was a subset of the participants previously reported on by Macbeth et al. (2022). In addition to English (the predominant community language), the participants knew a variety of other heritage languages. The heritage languages captured in the recordings (*n* included in parentheses) included Amharic (1), Arabic (1), Burmese (1), Cantonese (1), Farsi (2), Hindi (1), Igbo (1), Korean (3), Mandarin (6), Portuguese (1), Punjabi (1), Spanish (24), Teochew (1), Thai (1), and Vietnamese (6). Nine participants did not use their heritage language during the recording period. All participants were exposed to their HL from birth and acquired English between birth and age 12 years (*M* = 3.57 years). Moreover, the majority of participants reported their HL being the language they used (76.67%) and heard (88.33%) the most during their childhood prior to entering elementary school. The study was advertised through the psychology department’s participant pool. Participants were given $25 and course credit for their participation.

#### Characteristics of participants’ language environments

Participants reported exposure to various languages in their community. Southern California is a linguistically diverse region of the United States, where heritage bilinguals may have the opportunity to be exposed to the community language, their HL, as well as other languages. Such exposure to linguistic diversity may not only provide an environment in which bilingualism is supported but may also provide linguistic experiences that shape language and cognition (Bice and Kroll, 2019; Atagi and Sandhofer, 2020). Just over half of the participants reported hearing two or more languages in the communities in which they currently reside (53.33%), with 45% of participants hearing one or more languages other than English and their HL in their current communities. Additionally, 75% of participants reported hearing two or more languages on the campus of the university they currently attend, with 60% of participants hearing one or more languages other than English and their HL on campus. Moreover, 53.33% of participants reported hearing two or more languages in the communities in which they grew up, with 48.33% of participants reporting that they heard one or more languages other than English and their HL in those childhood communities. This retrospective self-reported data was corroborated by U.S. census data: Searching census data using the ZIP codes of the residences at which participants spent the majority of their childhood, we found that–on average–participants grew up in communities in which only 45.60% (*SD* = 20.66%, range: 8.80–89.50%) of the population spoke only English (U.S. Census Bureau, 2018). Thus, the heritage bilinguals in this sample not only had exposure to a HL in their homes, but also were exposed to various languages in their communities.

### Materials

#### Language background questionnaire

This in-house questionnaire combined a variety of questions from the Language History Questionnaire (Li et al., 2014) and the LEAP-Q (Marian et al., 2007), and assessed various aspects of language history and current language use. Participants provided demographic information and reported proficiency in both English and their HL on a scale of 1–7 (1 = Very poor, 7 = Native-like; e.g., “Please rate your current ability in speaking, reading, writing, and understanding in each language”), their age of acquisition (AoA) of each language (e.g., “For each language, enter an age for when you first became exposed to this language”), and their current exposure to and use of each language on a scale of 0–10 (0 = Never, 10 = Always, e.g., “Please rate how much you are currently using each language”). Participants were also given four scenarios adapted from the Bilingual Switching Questionnaire (Rodriguez-Fornells et al., 2012). Code-switching frequency was measured on a scale of 1 (Never) to 5 (Always).

#### Laboratory tasks

##### Verbal fluency

Participants were presented with a category (e.g., vegetables) on a computer screen and asked to name as many examples of that category as they could within 1 min. Category names were always presented in English, but on half of the trials, participants provided examples of the category in their HL. After a practice trial (colors), participants completed two blocks consisting of four English trials and four HL trials. The categories of clothing, drinks, sports, and vegetables always appeared together in the same block (Block A), and the categories of furniture, modes of transportation, fruits, and words associated with the beach were always presented in the same block (Block B), though the categories appeared in a random order for each participant. The blocks were counterbalanced such that half of the participants received Block A first, and half received Block B first. Further, half of the participants completed Block A in English, and half completed Block A in their HL, and then Block B was completed in the language that was not used for Block A. A total score for each language, a proxy of language proficiency, was created from the sum of all valid responses given for each of the four categories. Similar procedures have been used in past studies (e.g., Gollan et al., 2002; Linck et al., 2009; Baus et al., 2013).

##### Multilingual naming test

In the MINT (Gollan et al., 2012), pictures of objects were presented on a computer screen one at a time and remained until the participant made a verbal response, or a maximum of 5 s. Participants were instructed to say the name of each object in English, or if they did not recognize or know the name of the object, to say, “I do not know.” Most participants who identified as Spanish-English bilinguals also completed the MINT in Spanish (*n* = 18). There were five practice trials and 68 test trials. The total score on the test trials has been used in previous research as a measure of language proficiency and vocabulary knowledge (e.g., Tao et al., 2015).

#### The EAR

The “EAR on Android” app was downloaded from the Google Play store onto Motorola Moto E 2nd generation phones. Settings such as recording duration (length of recording) and interval (time between recordings) can be manually adjusted by the experimenter. In the current study, the EAR was set to record for 40 s every 12 min, with a six-hour blackout period at night based on when participants self-reported their typical bedtime. A more detailed description of the EAR and its utility for psycholinguistics-related research can be found in Macbeth et al. (2022).

### Procedure

Data was collected in two waves. While the procedures across both waves were quite similar, the laboratory tasks (verbal fluency and MINT) were only completed by participants in Wave 2 (*n* = 38). The Spanish MINT was only completed by a Spanish-speaking subset (*n* = 18) of these Wave 2 participants. The Language Background Questionnaire and stimuli for verbal fluency and the MINT were presented on a Dell Precision 3,420 computer running Windows 7 Professional, and recordings of verbal responses on verbal fluency and the MINT were captured using a Marantz Professional PMD-561 handheld recorder. Visual stimuli for verbal fluency and the MINT were presented *via* E-Prime 2.0, and questionnaire data was collected through Qualtrics. All instructions, tasks, and questionnaires were conducted in English, except for the HL verbal fluency and Spanish MINT trials. For HL verbal fluency, instructions and category cues were given in English, and participants were asked to respond in their HL. For the Spanish MINT, instructions were presented in Spanish. Participants came to the lab for two data collection sessions, one before and one after the 4 days of EAR audio recording.

#### Session 1

Participants were informed about the nature of the study, what types of sounds the EAR is designed to record, and information about the recording duration and interval. They were asked to wear the EAR as much as they were comfortable with, including locations such as home, school, in class, and other public places like a park or mall. The only location participants were told to not wear the EAR was at work, to avoid potential conflict with employers.

After confirming understanding of the recording procedures, participants were consented. They then completed the MINT. The EAR was programmed to begin recording immediately after the end of the testing session, and the recordings ended when the participant went to bed or at midnight on the fourth day of recording, whichever came first.

#### Interim recording period

For 4 days, either from Thursday–Sunday or Friday–Monday, the participants went about their daily lives while wearing the EAR. They could choose to wear the EAR either clipped to their waist or in an armband. Participants were, in general, quite compliant (Macbeth et al., 2022) and wore the EAR during approximately 80% of their waking hours.

#### Session 2

Participants returned the EAR on the day after recording was completed. While their audio files were uploaded to a secure server, participants completed a series of questionnaires including the Language Background Questionnaire. Following the self-report measures, participants completed the verbal fluency task. Once finished, participants were debriefed and compensated for their time and participation.

#### Data coding

##### The laboratory tasks

Participants’ responses on verbal fluency and the MINT were audio-recorded and later coded in the laboratory by research assistants who were heritage speakers of those languages. For example, Spanish-English heritage bilingual research assistants coded Spanish MINT data. Moreover, because research assistants who worked on this study came from the same, linguistically diverse university from which participants were also recruited, all HL verbal fluency were coded by research assistants who were also heritage bilinguals of those HLs.

##### The EAR

Audio files were coded by at least two research assistants who spoke the languages contained in the audio files. Due to privacy and ethical considerations, only participants’ speech—*not* conversation partners’ speech—was examined. Detailed descriptions of coding procedures, as well as ethical considerations, can be found in Macbeth et al. (2022).

We coded the proportion of audio files in which participants spoke their HL out of their total number of audio files with speech. This proportion serves as an approximate measure of the amount of time engaged in day-to-day HL use and will be referred to hereafter as “EAR-based HL use.” Additionally, we coded the proportion of files in which participants code-switched, defined here as speaking both English and HL within the audio file, out of their total number of files with speech. Although this measure of code-switching does not make fine-grained distinctions among different types of code-switching, this measure captures instances of both inter-and intrasentential code-switching and serves as an approximate measure of code-switching frequency; this measure will be referred to hereafter as “EAR-based code-switching.”

We chose to use the proportion of audio files with HL speech for each participant rather than the raw number of audio files with HL speech because we previously found that though the two values are strongly correlated, proportional, rather than absolute, predictors had greater variability which made them statistically more effective predictors (Macbeth et al., 2022). We chose to use file counts rather than word counts because these two values are also strongly correlated, but file counts allow researchers the option to forgo data transcription (a time-consuming process) and instead only code the language spoken in audio files. Only coding the language spoken in files also means that researchers do not have to develop a standard system of counting words across the various heritage languages that may be recorded by the EAR - this is beneficial since word boundaries vary across languages, and concepts may be represented by differing numbers of words across different languages (Macbeth et al., 2022).

## Results

### Sample characteristics

Table 1 reports descriptive statistics related to self-reported proficiency, AoA, and current use of the participants’ two languages. Participants were generally English dominant or balanced bilinguals, and on average, they learned their HL at a significantly younger age than they did English.^2^ They reported greater use of English in their day-to-day lives.

**Table 1:** Means, standard deviations, and *t*-tests for self-report items from the language background questionnaire, scores on laboratory-based proficiency tasks, and EAR-based measures.

	English		Heritage language		
	Mean *(SD)*	Range	Mean *(SD)*	Range	*t*-value
**Self-Report Items**					
Overall Proficiency	6.73 *(0.53)*	5–7	5.43 *(1.31)*	3–7	6.66*
Speaking	6.75 *(0.63)*	4–7	5.83 *(1.11)*	3–7	5.39*
Reading	6.78 *(0.49)*	5–7	5.02 *(2.04)*	1–7	6.32*
Writing	6.52 *(0.81)*	4–7	4.55 *(2.03)*	1–7	6.42*
Understanding	6.85 *(0.44)*	5–7	6.33 *(0.88)*	4–7	4.31*
Age of Acquisition	3.57 *(2.89)*	0–12	0.94 *(1.39)*	0–5	7.13*
Language Use	9.58 *(1.25)*	3–10	6.20 *(2.44)*	1–10	9.96*
**Laboratory Tasks**					
Verbal Fluency	50.61 *(12.45)*	25–87	29.91 *(11.76)*	10–72	7.53*
MINT	57.16 *(5.42)*	37–64	32.61^†^ *(10.32)*	16–53	9.97*
**EAR-Based Measure**					
Proportion of Audio Files	0.92 *(0.19)*	0.13–1.00	0.16 *(0.23)*	0.00–0.98	14.25*

Proficiency was rated on a scale of 1–7. Overall Proficiency is the average of self-reported proficiency ratings for speaking, reading, writing, and understanding. Age of acquisition was reported in years. Rates of one’s language use were rated on a scale of 0 (never)-10 (always). Proportion of audio files reflects the proportions of the number of audio files containing speech in each language out of the total number of audio files with speech. All comparisons between English and HL were statistically significant.

* *p* < 0.001.

† Indicates that the HL MINT only includes scores from participants who completed the MINT in Spanish (*n* = 18). The corresponding t-test only compares the English and Spanish scores from those 18 participants.

Self-reported code-switching was also fairly common. In general, participants rated themselves as moderate switchers during conversations (*M* = 3.20, *SD* = 1.07), in certain situations (*M* = 3.57, *SD* = 1.08), when discussing certain topics (*M* = 3.07, *SD* = 1.30), and also as moderate language mixers (*M* = 3.28, *SD* = 1.20).

Additionally, the laboratory measures of language proficiency were consistent with the self-report measures in that participants were highly proficient in English (see Table 1). Participants consistently performed better, on average, on the verbal fluency and MINT tasks in English compared to their HL.

There was variability in EAR-based measures of language use. Participants produced an average of 208.15 audio files (*SD* = 61.64, range = 44–318), of which an average of 75.37 audio files contained speech (*SD* = 33.65, range = 15–169). Significantly more audio files contained English speech (*M* = 71.23, *SD* = 36.09, range = 2–168) than speech in the HL (*M* = 9.63, *SD* = 13.41, range = 0–88), and very few audio files contained code-switching (*M* = 5.50, *SD* = 6.23, range = 0–25)—that is, audio files that contained both English and the HL speech within a single audio file.

As captured by the EAR, our sample spoke in 37.1% (*SD* = 13.7%, range = 8.2–76.1%) of their valid recorded audio files, on average. A file was considered valid if the participant was awake and wearing the EAR during the recording. Participants used their HL in 15.9% (*SD* = 22.9%, median = 7.7%, range = 0–97.8%) of speech files, and code-switching occurred in 7.7% (*SD* = 8.5%, median = 4.9%, range = 0–31.0%) of speech files. Of our participants, 15.0% never used a HL during the recording period, and 23.3% never code-switched.

### Relationships between self-report, laboratory tasks, and EAR

First, we conducted a series of correlations to examine the zero-order relationships between our measures (see Table 2). The goal of these analyses was to better understand individual characteristics that contribute to variable use of the HL. A series of noteworthy relationships emerged. Among our three self-report variables, self-reported HL use was moderately and positively related to both self-reported HL overall proficiency—the average of self-reported proficiency ratings for speaking, reading, writing, and understanding—and English AoA. The more a heritage speaker uses their HL, the more proficient they report themselves to be in their HL. Further, the more a heritage speaker uses their HL, the older they were when they learned English, suggesting they likely had more sole exposure to, and use of, their HL prior to English being introduced. However, self-reported English AoA and HL overall proficiency were not related to each other, which suggests that the age at which one acquired English has less bearing on their reported HL proficiency.

**Table 2:** A correlation matrix showing the relationships between the self-report items, participants’ scores on laboratory tasks, and day-to-day HL use derived from the EAR.

	1. HL Ov Prof	2. HL Use	3. Eng AoA	4. HL VF	5. Eng MINT
**Self-Report Items**					
1. HL Overall Proficiency					
2. HL Use	0.42*				
3. English AoA	0.27	0.44*			
**Laboratory Tasks**					
4. HL Verbal Fluency	0.57*	0.46*	0.04		
5. English MINT	−0.26	−0.31	−0.29	−0.22	
**EAR-Based Measure**					
6. HL Use	0.39*	0.51*	0.43*	0.60*	−0.65*

*N* = 60 for all correlations except those with English MINT (*n* = 38) and HL verbal fluency (*n* = 35).

* Indicates significance at a Bonferroni-corrected value of *p* of 0.008.

Among our laboratory-based tasks, we focused on HL verbal fluency and the English MINT. While HL performance on the MINT would arguably be more interesting given the greater variability in scores as compared to English performance, we did not examine the Spanish MINT due to the small number of participants who were able to complete this task (*n* = 18). The relationship between HL verbal fluency and English MINT was not significant, which suggests that a heritage speaker’s HL and English abilities are independent of one another.^3^ Interestingly, HL verbal fluency was related to self-reported HL overall proficiency, suggesting that heritage speakers are fairly, though not perfectly, accurate at assessing their skill in their HL. Further, HL verbal fluency was also related to self-reported HL use, indicating that the objective ability one displays in their HL appears to predict how much one uses the HL.

**Figure 1:**
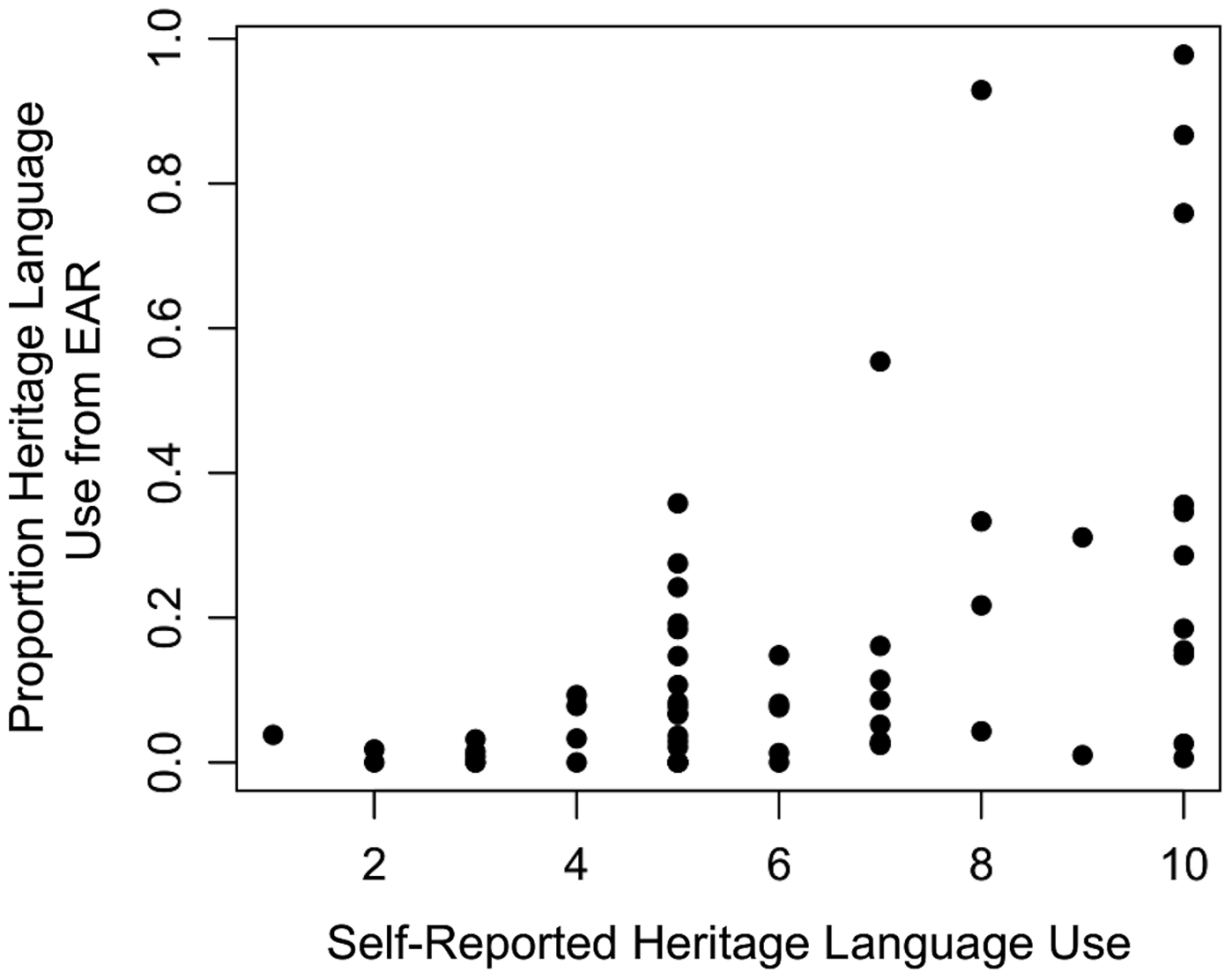
Scatterplot depicting the relationship between EAR-based and self-reported heritage language use.

EAR-based HL use was significantly correlated with all the other self-report items and laboratory tasks. Of the self-report items, the strongest relationship existed between EAR-based HL use and self-reported HL use, as shown in Figure 1. Our self-report measure of HL use asked participants to rate how often they used their HL on a 0–10 scale from “never” to “always” so these ratings cannot be interpreted as proportion of time spent speaking, or proportion of utterances produced in the HL. Though the absolute self-report values may not map on to the utterance proportion values derived from the EAR, participants’ self-report values nonetheless provide a useful assessment of the prevalence of the HL in their lives. We expect that individuals who self-report higher values have greater exposure to their HL and individuals who report lower values have less exposure. The correlation between self-reported and EAR-based HL use is noteworthy because while both assessments claim to be measuring a similar construct, only about 25% of the variance in one variable is accounted for by the other. We see some, though imperfect, alignment between self-reported and EAR-derived estimates of HL use.

Interestingly, it is evident from Figure 1 an asymmetry between self-reported and EAR-based language use emerged. Those who self-reported infrequent use of the HL tended to indeed use that language infrequently relative to other participants, but there was a great deal of variability among individuals who self-reported frequent use of the HL. These participants may have used the HL frequently, but they also may have hardly used it at all, as evidenced by the large vertical spread of scores on the right side of Figure 1. While the EAR might not have captured all possible instances of the participants using (or not using) their HL, such findings suggest that self-report is not entirely reliable on its own as an assessment of language use, particularly for individuals who self-report high rates of HL use.

**Figure 2:**
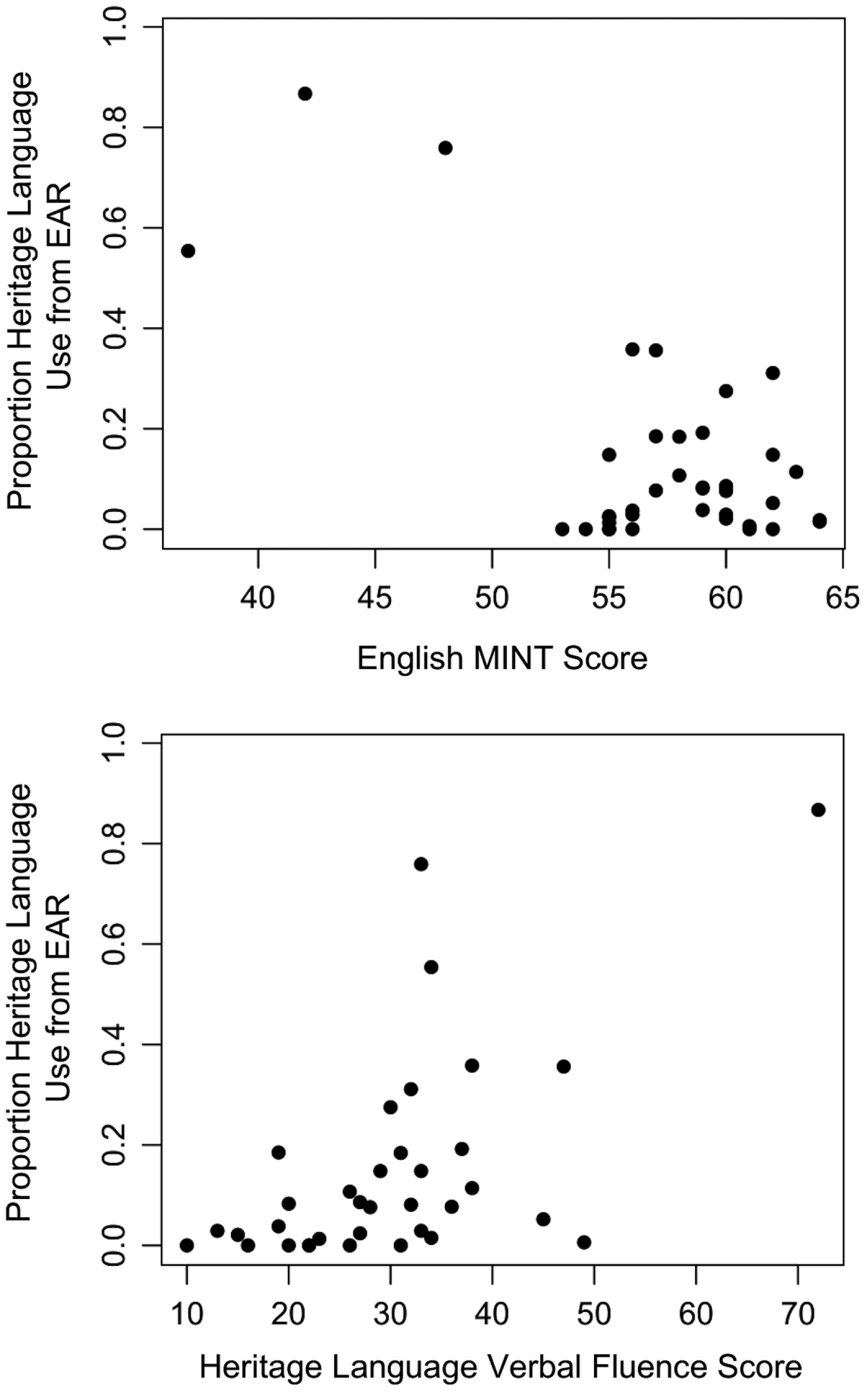
Scatterplots showing the relationships between EAR-based heritage language use and English MINT **(top)**, and EAR-based heritage language use and HL verbal fluency **(bottom)**. Outliers that were >3 SDs higher or lower than the mean were removed from further analysis.

EAR-based HL use was also strongly related to HL verbal fluency and English MINT, as shown in Figure 2. Participants who used their HL more often had higher HL verbal fluency scores and lower English vocabulary scores. Upon further examination of the data, there was one participant with a HL verbal fluency score greater than 3 standard deviations above the mean, and one other participant with an English MINT score greater than 3 standard deviations below the mean. After removing these two participants from the analysis, both the relationship between EAR-based HL use and HL verbal fluency as well as the relationship between EAR-based HL use and English MINT were weakened. These results suggest that HL verbal fluency and laboratory-based English proficiency are moderately correlated with EAR-based HL use. For the remainder of the analyses, HL verbal fluency and English MINT do not include these two outlier data points.

### Predicting EAR-based HL use from self-report

The correlation matrix in Table 2 showed that multiple self-report items correlated with each other and with EAR measures of actual language use, so we aimed to better understand whether these correlations account for different or shared sources of variance. Because not all participants completed the laboratory tasks, we were first interested in the effects of self-report variables on their own, across the entire sample, prior to examining models that included all three instruments. For example, if both self-reported HL use and overall proficiency predict EAR-based HL use, do these two predictors account for similar variability within EAR-based HL use? Likewise, to what extent could one consider the two assessments to capture similar variance such that they could hypothetically be used interchangeably? To answer these questions, we conducted a multiple regression to determine which of these self-reported language background variables best predicted EAR-based HL use. All variables were *z*-scored prior to being entered in the model.

The simultaneous regression model included self-reported HL overall proficiency, HL use, and English AoA as predictors of EAR-based HL use. The model was significant, *F*(3, 56) = 9.72, *p* < 0.001, R^2^ = 0.34, with only self-reported HL use serving as a significant predictor of EAR-based HL use, as shown in Table 3 (although English AoA was marginally significant). In this model, much of the variance in EAR-based HL use is left unexplained by self-report, suggesting that the EAR is providing substantial unique information about real-world language use of heritage bilinguals that self-report is unable to capture.

**Table 3:** Multiple regression analysis predicting EAR-based heritage language use from self-reported language background variables.

Self-Report Predictors	B	*t*-value	*p*-value	*r*	*r*_partial_
HL Overall Proficiency	0.18	1.54	0.13	0.39	0.20
HL Use*	0.33	2.57	0.01	0.51	0.33
English AoA	0.24	1.96	0.06	0.43	0.25

Significant predictors are noted with an asterisk (*).

Further, upon examining the partial correlation for each predictor (see Table 3), there is substantial shared variance among the self-report items. Without self-reported HL overall proficiency and English AoA, the relationship between self-reported HL use and EAR-based HL use grows much weaker, going from *r* = 0.51 to 0.33. The amount of HL use by a given individual appears to be partly predicted by how proficient they are in their HL, as well as when they began learning English. Thus, while self-report is overall not an entirely reliable means of capturing current language use, using multiple items to predict real-world language use appears to be beneficial.

### Examining shared variance among self-report, laboratory tasks, and the EAR

Finally, we wanted to examine which predictors were contributing unique or overlapping variance among all three of our language assessments. For these analyses, we focused on our smaller subsample that completed the laboratory tasks in addition to the self-report and EAR portions of the study (*n* = 35). Our hope was to provide further insight on what self-report, laboratory tasks, and the EAR tell us individually, and as a set, in the context of understanding the language experiences of heritage bilinguals. Self-reported HL overall proficiency, HL use, and English AoA were again the self-report variables of interest, HL verbal fluency was used as the laboratory-based variable of interest, and EAR-based HL use was our experience-based variable. The English MINT was not considered in these analyses because there was more variability in HL verbal fluency scores, and we were ultimately more interested in the relationships between self-reported and lab-based HL proficiency measures.

We used a series of simultaneous regression models to examine the shared and unshared variance among our constructs (see Table 4). The first three regression models examined how well HL verbal fluency and EAR-based HL use predicted each of our three self-reported outcome variables. The other self-report items were not included as predictors in the first three models (e.g., self-reported English AoA and HL use were not included as predictors of self-reported HL overall proficiency), because our research questions were inherently more focused on shared variance between assessments as opposed to shared variance among items on a single assessment. In Model 1, we found that HL verbal fluency significantly predicted self-reported HL overall proficiency, but EAR-based HL use did not, *F*(2, 31) = 8.43, *p* = 0.001, R^2^ = 0.35. These findings suggest that a heritage bilingual’s lab-based proficiency is consistent with how they self-report their HL proficiency. Further, the lack of relationship between HL verbal fluency and EAR-based HL use makes sense; the EAR measure of HL use is not a measure of language proficiency but rather day-to-day language use. It is not necessarily the case that the most proficient HL speakers tend to use their HL most frequently. Day-to-day language use and language proficiency are not the same construct and may not even be strongly related.

**Table 4:** Multiple regression analyses examining sources of shared and unshared variance between different predictors.

Predictor	*B*	t-value	*p*-value	r	*r*_partial_
**Model 1: Self-Reported HL Overall Proficiency**					
HL Verbal Fluency*	0.54	3.05	0.005	0.56	0.48
EAR-Based HL Use	0.27	1.46	0.15	0.40	0.25
**Model 2: Self-Reported HL Use**					
HL Verbal Fluency	0.27	1.41	0.17	0.37	0.25
EAR-Based HL Use*	0.49	2.37	0.02	0.47	0.39
**Model 3: Self-Reported English AoA**					
HL Verbal Fluency	−0.27	−1.40	0.17	0.06	−0.24
EAR-Based HL Use*	1.00	5.02	< 0.001	0.64	0.67
**Model 4: HL Verbal Fluency**					
Self-Reported HL Overall Proficiency*	0.40	2.90	0.007	0.56	0.47
Self-Reported HL Use	0.19	1.35	0.19	0.37	0.24
Self-Reported English AoA*	−0.29	−2.06	0.05	0.06	−0.36
EAR-Based HL Use	0.34	1.65	0.11	0.36	0.29	**Model 5: EAR-Based HL Use**
Self-Reported HL Overall Proficiency	0.04	0.29	0.78	0.40	0.05
Self-Reported HL Use	0.07	0.60	0.56	0.47	0.11
Self-Reported English AoA*	0.41	3.82	< 0.001	0.64	0.58
HL Verbal Fluency	0.25	1.65	0.11	0.36	0.29

The outcome variable for each regression model is bolded with predictors listed below. Significant predictors for each regression model are noted with an asterisk (*).

In Models 2 and 3, only EAR-based HL use significantly predicted self-reported HL use, *F*(2, 31) = 5.74, *p* = 0.01, R^2^ = 0.27, and English AoA, *F*(2, 31) = 12.67, *p* < 0.001, R^2^ = 0.45, while HL verbal fluency was not a significant predictor in either model. This implies that heritage bilinguals’ lab-based proficiency in their HL is not a predictor of their self-reported HL use, nor is it a predictor of the estimated age at which they acquired English. In this case, an experience-based measure of day-to-day language use is a better predictor of participants’ estimate of their self-reported HL use and perhaps more surprisingly, AoA. In fact, it appears that in the model predicting English AoA, the zero-order relationship between English AoA and EAR-based HL use was statistically suppressed, as evidenced by the partial correlation between the two variables. This suggests that controlling for some of the random variability in other variables within the regression model, such as HL verbal fluency, actually strengthens the relationship between English AoA and actual, EAR-based HL use.

Next, we sought to determine which self-report and experience-based variables best predicted HL verbal fluency scores. Model 4 shows that two self-report variables, HL overall proficiency and English AoA, are significant predictors of HL verbal fluency, *F*(4, 29) = 5.47, *p* = 0.002, R^2^ = 0.43, while self-reported HL use and EAR-based HL use are not significant predictors. Again, this makes sense because day-to-day HL use, whether it is assessed through self-report or the EAR, is not the same underlying construct as language proficiency. Again, we see a dissociation between current day-to-day language use and measures of language proficiency such that it is not necessarily the most proficient speakers who currently use their heritage language most often. Therefore, if researchers are interested in approximating a participant’s language abilities from self-report, the participant’s self-reported proficiency and AoA may be the most indicative items. Interestingly, suppression was also present in this model for English AoA, which implies that the relationship between verbal fluency and AoA is typically obscured by the shared variance between AoA and other self-report items.

Finally, Model 5 predicted EAR-based HL use from self-report and HL verbal fluency. English AoA was the only variable to significantly predict EAR-based HL use, *F*(4, 29) = 8.10, *p* < 0.001, R^2^ = 0.53. Importantly, self-reported HL use was not an important predictor of EAR-based HL use, even though the opposite was true in Model 2 (see Table 4). This suggests that much of the variance in self-reported HL use and English AoA is shared, with the partial correlation between self-reported HL use and EAR-based HL use growing much weaker (from *r* = 0.47 to 0.11) after controlling for English AoA and the other proficiency-related variables. Therefore, if a heritage bilingual has a good estimation of when they began speaking their non-heritage language (English in this case) and if other sources of variance (e.g., proficiency, use) are also accounted for, AoA may relate most strongly to day-to-day HL use, if such experience-based measures such as the EAR are not feasible or available.

### Exploratory code-switching analyses

Next, as an exploratory measure, we examined how well self-reported code-switching frequency captured the variability in EAR-based code-switching. The self-reported tendency to switch during a conversation was significantly correlated with EAR-based code-switching, *r*(58) = 0.52, *p* < 0.001. In addition, switching more during particular situations, *r*(58) = 0.27, *p* = 0.04, switching more when discussing certain topics, *r*(58) = 0.30, *p* = 0.02, and mixing more frequently, *r*(58) = 0.30, *p* = 0.02 were also moderately related to EAR-based code-switching. When these four variables (z-scored) were entered into a simultaneous regression model, the model significantly predicted EAR-based code-switching frequency, *F*(4, 55) = 5.24, *p* = 0.001, R^2^ = 0.28. However, the self-reported tendency to switch during a conversation was the only significant predictor of EAR-based code-switching (*B* = 0.60, *p* < 0.001). Therefore, of the four self-reported code-switching questions, self-rated frequency of switching was selected as the item that best served as a proxy for actual EAR-based code-switching and is the predictor we use and report in subsequent analyses.

We also wondered how frequency of HL use related to code-switching frequency. In other words, if someone uses their HL a lot, are they more likely to code-switch, or are these two aspects of language use independent? Interestingly, the relationship between self-reported HL use and self-reported code-switching frequency [*r*(58) = 0.30, *p* = 0.02] as well as the relationship between EAR-based HL use and EAR-based code-switching [*r*(58) = 0.60, *p* < 0.001] were both significant. Together, these findings suggest that participants who use their HL more often also code-switch more frequently. However, it is possible that the EAR may provide better estimates of “true” code-switching frequency compared to self-report: Participants may not be fully aware of how much or how little they code-switch and have a harder time estimating that for themselves. It should be noted that like the relationship between EAR-based HL use and self-reported HL use, EAR-based measures of code-switching frequency reflect a true proportion of utterances containing code-switching, whereas self-reported code-switching frequency is a rating scale that may not directly map onto the true proportion of code-switched utterances as measured by the EAR.

We then asked whether any other self-report or laboratory measures of language proficiency that we examined previously (e.g., self-reported HL proficiency, HL use, English AoA, or HL verbal fluency) would aid in predicting EAR-based code-switching frequency. The regression model was significant, *F*(5, 28) = 9.73, *p* < 0.001, R^2^ = 0.64. In addition to self-reported code-switching frequency, English AoA and HL verbal fluency were also significant predictors of EAR-based code-switching (see Table 5). Neither self-reported HL proficiency nor self-reported HL use were predictors of EAR-based code-switching. These results suggest that, in general, how well a bilingual believes they know one of their languages or how often they use it, is independent of switching frequency. However, acquiring English at an older age and being more proficient in the HL are associated with more frequent code-switching.

**Table 5:** Multiple regression analysis predicting EAR-based code-switching frequency from self-report and laboratory-based variables.

Predictor	*B*	*t*-value	*p*-value	*r*	*r*_partial_
Self-Reported Code-Switching*	0.50	4.19	<0.001	0.63	0.58
Self-Reported HL Proficiency	−0.10	−0.62	0.54	0.31	−0.12
Self-Reported HL Use	−0.02	−0.15	0.89	0.38	−0.03
Self-Reported English AoA*	0.43	3.14	0.004	0.55	0.51
HL Verbal Fluency*	0.45	2.38	0.03	0.41	0.41

Significant predictors are noted with an asterisk (*).

## Discussion

The goals of this study were threefold: to examine the relationships among three different measures of a heritage bilingual’s language background, to determine how well self-report measures predict real-world HL use, and to investigate the extent to which various self-report items, laboratory tasks, and objective assessments of day-to-day language use “hang together” and serve as mutually predictable information about a heritage bilingual’s linguistic experiences. Further, we were also interested in how well heritage bilinguals can gauge their own frequency of code-switching, and whether other measures of bilingual proficiency or use can aid in predicting real-world code-switching tendencies above and beyond assessing code-switching through self-report.

We generally found moderate to strong relationships between the self-report items, laboratory tasks, and EAR-based measure of HL use, suggesting that they are all assessing similar, though not entirely overlapping, constructs. An interesting trend that emerged was that in reflecting upon one’s own language use, many heritage bilinguals tended to overestimate *via* self-report how frequently they used their HL, in comparison to how frequently they actually used their HL as measured by the EAR.

These findings are in line with past studies that have shown that young adults tend to show an enhancement bias (MacIntyre et al., 1997; Gollan et al., 2012), which might lead to overestimation of their HL proficiency and by proxy, their HL use too. Further, nearly half of our sample consisted of Spanish-English bilinguals, who have been shown to be less accurate in self-rating their proficiency compared to other cultural groups (Sheng et al., 2014), particularly when they are less proficient in Spanish compared to English (Tomoschuk et al., 2019). Overestimation of the less dominant language–in the case of the present study, the HL–is also consistent with past work by Marchman et al. (2017).

Our second set of results demonstrated that out of three commonly used self-report items (overall proficiency, frequency of language use, and AoA), self-reported frequency of HL use was a significant predictor—and English AoA, a marginally significant predictor—of day-to-day EAR-based HL use. However, when examining our simultaneous regression model involving all three measures (self-report, laboratory, and EAR-based measures), it was evident that English AoA accounted for the most unique variance of EAR-based HL use, above and beyond self-reported HL use. These two models likely yielded different results due to the inclusion of the laboratory task (i.e., HL verbal fluency) in the latter model: HL verbal fluency was significantly correlated with both self-reported overall proficiency and self-reported HL use, likely “soaking up” the variance associated with those two self-reported measures. Thus, among heritage bilinguals, the age at which one acquired the majority, community language (in this case, English) appears to be particularly important in understanding the frequency with which one uses their HL in everyday life.

It is intuitive that we found older English AoA to be coupled with more frequent use of the HL. The heritage bilingual likely had greater practice with the HL and greater exposure across their lifetime to other speakers of their HL. Moreover, there may be characteristics of heritage bilinguals who are later-learners of the community language that are associated with more frequent HL use (e.g., their family members may speak the community language less). This may be one reason why we found English AoA—rather than self-reported HL use—to be a unique predictor of real-world HL use. English AoA in this study may be indexing aspects of a heritage bilingual’s language history that is not captured in other self-report measures but is relevant for how the heritage bilingual currently uses their HL. For example, English AoA here may be tapping into the ways in which a heritage bilingual’s family uses English and the HL: If family members used the HL more frequently in the past, it is possible that the HL may still be spoken more frequently with family members. Such language history characteristics may not be captured by self-reports of current language proficiency or use. Another reason might be that self-reporting one’s AoA is more objective than self-reporting HL use. When asked about AoA, a bilingual may be able to recall some milestone in their lives associated with acquisition of the given language (e.g., immigrating to a new country or starting school). However, it is arguably more difficult to gauge the frequency with which you use a language because there are many situational or contextual factors (e.g., who you are with, where you are, or what you are doing) that may influence the amount of a language used on any given day. This may be another reason why the frequency of HL use is difficult to self-report. Therefore, AoA may be easier for participants to report and may capture other characteristics of heritage bilinguals’ language experiences that make AoA an informative indicator of real-world language usage.

Turning to measures of proficiency, self-reported HL proficiency and HL verbal fluency appear to be mutually predictive, and one’s self-reported proficiency in their HL was not indicative of how often they used the HL day-to-day. These results suggest that proficiency and frequency of use of a given language are separable constructs and largely independent of one another, a finding that is consistent with past research (Gollan et al., 2015). Just because someone is highly proficient in a HL–perhaps as a function of past immersion or exposure–does not mean that the language is being used often in the current context being captured. On the other hand, while previous studies have posited that laboratory-based measures are not the best assessments of linguistic skill (Friesen et al., 2015; Paap et al., 2017), our results with heritage bilinguals are in line with other studies (e.g., Marian et al., 2007; Shi, 2011, 2013) that show that self-reported language proficiency and laboratory measures of language proficiency are moderately correlated and may explain similar variation in language proficiency.

With regard to the exploratory code-switching analyses, only the self-report item that asked participants about their frequency of code-switching during a conversation significantly predicted EAR-based code-switching. It seems that self-report items which ask about the contextual aspects of code-switching, such as whether particular situations or topics may induce more code-switching, do not predict EAR-based code-switching frequency above and beyond what self-reported conversational switching frequency tells us. It is not clear whether the poor predictive power of the contextual or situational effects of code-switching stem from participant’s challenge to accurately report these behaviors, or if these contextual and situational effects genuinely do not relate to overall code-switching frequency. Additionally, it should be noted that the measure of EAR-based code-switching frequency reported here was a proportion of audio files containing speech in both English and the HL in a single audio file. It is therefore possible that a more fine-grained analysis of EAR-based code-switching (e.g., examining transcriptions of code-switched speech) may show real-life code-switching to be predicted by self-report items about the contextual or situational aspects of code-switching.

Another interesting result that emerged from the code-switching data was the finding that EAR-based code-switching and EAR-based HL use were more strongly related to one another than self-reported code-switching and self-reported HL use. Such findings support past work suggesting that bilinguals are often not aware of when they code-switch (Gumperz, 1982), which may be influencing the strength of the relationship between self-reported code-switching and self-reported HL use. Since the EAR-assessed measures of code-switching and HL use are arguably more objective, these results suggest that self-reported code-switching frequency might not be a strong proxy for real-world code-switching, and that the EAR may be more accurate at gauging such behavior.

Further, we found that English AoA and HL verbal fluency predicted code switching such that later English AoAs and higher HL verbal fluency scores were associated with more code-switching. Both later English AoA and higher HL verbal fluency scores are associated with greater proficiency in the HL. The finding that HL proficiency *positively* predicts real-world code-switching is consistent with work emphasizing that code-switching is used by bilinguals who are highly proficient in their two languages, rather than individuals who lack skill in one or both languages (Poplack, 1980). In fact, some argue that for highly proficient bilinguals, one’s two languages are so integrated that code-switching becomes an opportunistic, almost effortless process (Green and Abutalebi, 2013). Our naturalistic data provides converging information that code-switching is associated with highly skilled language use. All participants in our sample were highly proficient English speakers, and greater proficiency in the HL was associated with higher rates of code-switching.

Given the effectiveness of the EAR as a tool to assess frequencies of HL use, could the naturalistic speech samples collected by the EAR be used to assess language proficiency as well? While this may be possible, some challenges arise. As we describe in past work (Macbeth et al., 2022; cf. Montag et al., 2018), given boundaries associated with forming sensible natural language sentences (e.g., function words must appear alongside content words), there is remarkably little variability in the lexical diversity of participant speech as captured by the EAR. At the sample sizes at which the EAR is typically used, there is not nearly enough meaningful variability in lexical diversity for it to be useful measure of vocabulary size or other aspects of word use. Hypothetically, researchers could code speech for various types of errors, but error rates may be quite low, again at the sample sizes typically collected. Likewise, researchers could hypothetically code utterances for syntactic complexity but given the rarity of complex syntax in spoken relative to written language (e.g., Biber, 1988) and the small sample sizes of speech, this method also may not yield stable estimates of complex language use. We are certainly open to the idea that naturalistic speech samples might be used to compute measures of language proficiency, so long as researchers avoid clear pitfalls associated with limits on spoken language lexical diversity and various consequences of the small size of EAR speech samples.

## Limitations and future directions

As with any study, the work reported here is not without limitations. The laboratory tasks–verbal fluency and the MINT–were not added to the study protocol until partway through data collection, resulting in a smaller sample for those measures compared to the self-report and EAR assessments. Because of this, we could not reliably examine the relationships between HL (Spanish) MINT scores with other variables of interest. Future work would benefit from examining variability in laboratory-based language measures in heritage bilinguals. For example, in addition to Spanish, the MINT has also been normed in other non-English languages such as Mandarin and Hebrew (Gollan et al., 2015). For studies examining English learners, measures such as LexTALE (Lemhöfer and Broersma, 2012) may also be a valid measure of language skill in select languages, such as Dutch and German. Beyond such lexical tasks, including laboratory-based measures of morphosyntax (e.g., grammaticality judgment tasks), phonology (e.g., phonemic discrimination tasks), semantics (e.g., semantic relatedness judgment tasks), and/or pragmatics (e.g., perspective taking tasks) may provide a more detailed account of language proficiency than lexical measures alone. Using such measures would allow for deeper investigations of the relationships between non-English performance in these laboratory-based language measures, self-ratings, and EAR-based language use.

While the EAR methodology provides an important window into the day-to-day linguistic experiences of bilinguals, certain limitations exist with naturalistic data collection. First, one of the most appealing aspects of the EAR, that it captures an intermittent sample of language use, can also mean that certain linguistic characteristics might be missed if they are not occurring frequently. Further, EAR can only capture spoken language use, and in today’s digital world, much communication is written. An undergraduate heritage bilingual might text or email in a HL, another aspect of real-world HL use, but this cannot be captured *via* audio recording. It is unclear whether rates of HL speech among heritage bilingual undergraduates would match the rates of HL text they produce day-to-day, but this would be an interesting avenue to pursue.

There are also limitations to the aspects of language history that our survey items were designed to assess. The items on our language background measure were primarily drawn from the LEAP-Q (Marian et al., 2007) and LHQ (Li et al., 2014). However, our measure did not capture fine-grained information about non-English language use in the home and in various social settings like school and religious activities (cf. LSBQ; Anderson et al., 2018). It is possible that capturing such information would have allowed us to explain more of the variability in real-world language use examined in the present study. Further, the EAR is capable of providing researchers with information about the number of audio files with speech in different contexts, since it is fairly easy to discern where a participant is and what they are doing throughout the recording period. Future work using the EAR and LSBQ in tandem could yield interesting findings regarding frequency of language use in more specific contexts and with specific interlocutors.

Additionally, we encourage replication and expansion of this study methodology with other bilingual populations. The undergraduate sample of Southern California heritage bilinguals used in the present study differs from other bilingual populations in many ways, the most important being that our sample was primarily English-dominant (i.e., dominant in the majority language), as evidenced *via* both self-report and lab-based proficiency measures. Future work should investigate how the conceptualization of proficiency among heritage bilinguals, as well as their patterns of language use, might differ from self-ratings or speech patterns produced by individuals who identify as more dominant in their HL.

## Conclusion

Overall, the results of this study suggest that each of the three measures examined in this study–self-report, laboratory-based tasks, and EAR-based assessments–capture some unique variability in the experiences of heritage bilinguals. For example, it is evident that the EAR provides an estimate of day-to-day language use that is just not possible to attain *via* self-report items or laboratory-based proficiency scores. As such, the EAR should be used for any study where researchers wish to sample naturalistic patterns of bilingual speech and understand how a bilingual’s languages are being used in the real world. Similarly, self-report and lab-based tasks have the benefit of being quick and easy to administer, appear to be fairly consistent with each other, and provide unique information about language proficiency that cannot be obtained using the EAR. Therefore, one or both of these measures should be used when information about an individual’s linguistic knowledge and abilities is paramount. While none of these measures strongly correlated–nor did we expect them to–it was evident that they capture information about the heritage bilingual language experience that is shared in some aspects and unique in others. We suggest they be used in tandem to yield the most important insights for the particular research questions being addressed.

## Data availability statement

The datasets presented in this study can be found in online repositories. The names of the repository/repositories and accession number(s) can be found at: The Open Science Repository (OSF): osf.io/mpjzy.

## Ethics statement

The studies involving human participants were reviewed and approved by the Institutional Review Board (Socio-Behavioral) at the University of California, Riverside. The participants provided their written informed consent to participate in this study.

## Author contributions

AM: primary author and editor of the manuscript, conducted data analysis, participated in project conceptualization and implementation, as well as overseeing coding and transcription of raw data. NA: contributed to writing of original manuscript as well as proofreading and editing, created supplementary materials. JM: contributed to proofreading and editing the manuscript, created figures. MB: participated in project conceptualization and implementation, as well as overseeing coding and transcription of raw data. CC: initial project conceptualization, provided the resources and supervision for project implementation, contributed to proofreading and editing the manuscript. All authors contributed to the article and approved the submitted version.

## Funding

This work was partially supported by a James S. McDonnell Foundation Scholar Award to JM, as well as a National Science Foundation Postdoctoral Research Fellowship under Grant No. SBE-1714925 and CSUF Junior Grant to NA.

## Conflict of interest

The authors declare that the research was conducted in the absence of any commercial or financial relationships that could be construed as a potential conflict of interest.

## Publisher’s note

All claims expressed in this article are solely those of the authors and do not necessarily represent those of their affiliated organizations, or those of the publisher, the editors and the reviewers. Any product that may be evaluated in this article, or claim that may be made by its manufacturer, is not guaranteed or endorsed by the publisher.

## Footnotes

^1^The LENA system is a digital language recording device designed to be worn by a young child that can record up to 16 h of the auditory environment. Unlike the EAR, which uses time sampling methods to record random samples of the auditory and linguistic environment, the LENA records continuously.

^2^The mean heritage language age of acquisition is not age 0; this reflects the fact that some participants self-reported simultaneously being exposed to their two languages at an age older than 0 (e.g., age 3). We left the data as is to reflect the participants’ perceptions of when they acquired their languages, even though it is highly unlikely that the individual received no language input until several years after birth.

^3^To double-check this assumption, we also correlated HL verbal fluency and English verbal fluency scores, and found no relationship (*r* = 0.12, *p* = 0.497), again suggesting that as a heritage bilingual, being proficient in one language does not guarantee better or worse proficiency in the other language. However, English verbal fluency scores and English MINT scores were significantly related (*r* = 0.50, *p* = 0.001), suggesting that both English verbal fluency and English MINT captured participants’ language skill in English.
